# Supplementary material for: Task-induced neural covariability as a signature of approximate Bayesian learning and inference
Source: PLoS Comput Biol. 2022 Mar 8;18(3):e1009557. doi: 10.1371/journal.pcbi.1009557 (PMC8963539; doi:10.1371/journal.pcbi.1009557)
Supplement: S1 Text — (PDF) [file pcbi.1009557.s004.pdf]

Task-induced neural covariability as a signature of approximate Bayesian learning and inference

Richard D. Lange[1,2,¤,*], Ralf M. Haefner[1,2,*],

1 Brain and Cognitive Sciences, University of Rochester, Rochester, New York, USA
2 Center for Visual Science, University of Rochester, Rochester, New York, USA

¤Current Address: Department of Neurobiology, University of Pennsylvania, Philadelphia, Pennsylvania, USA
* lange.richard.d@gmail.com (RDL), * ralf.haefner@rochester.edu (RMH)

S1 Text

Note on the apparent circularity of the ideal learning condition

Equation (2) defines the optimal task-prior (left hand side) in terms of the average posterior seen in the task (right hand side). Each posterior is, apparently circularly, defined in terms of the prior:

$$\begin{aligned}\mathrm{p_b}(\mathbf{x}|C) &= \mathbb{E}_{\mathrm{p_e}(s|C)}[\mathrm{p_b}(\mathbf{x}|s)] \\ &= \mathbb{E}_{\mathrm{p_e}(s|C)}\left[\sum_{C'} \frac{\mathrm{p_b}(\mathbf{x}|C')\mathrm{p_b}(s|\mathbf{x})}{\mathrm{p_b}(s)}\right] \quad .\end{aligned}$$

Equation (2) describes the *end result* of learning a task in terms of a fixed-point relation where the average posterior in the task is equal to the prior, but it does not prescribe how to arrive at such a prior.

A straightforward method to learn such a prior is to iterate until convergence, where in each step of the iteration, the "new" prior is defined as the average posterior under inferences made using the "old" prior:

$$\mathrm{p_b^{(t+1)}}(\mathbf{x}|C) = \mathbb{E}_{\mathrm{p_e}(s|C)}\left[\frac{\mathrm{p_b}(s|\mathbf{x})}{\mathrm{p_b^{(t)}}(s)} \sum_{C'} \mathrm{p_b^{(t)}}(\mathbf{x}|C')\mathrm{p_b}(C')\right] \tag{S1}$$

where we have assumed that it is only the prior influence of the category on the sensory representation $\mathrm{p_b}(\mathbf{x}|C)$, not the sensory generative procedure $\mathrm{p_b}(s|\mathbf{x})$ that changes with learning. It follows that the full prior on $\mathbf{x}$ $\mathrm{p_b^{(t+1)}}(\mathbf{x})$ is also defined iteratively as

$$\mathrm{p_b^{(t+1)}}(\mathbf{x}) = \mathbb{E}_{\mathrm{p_e}(s,C)}\left[\frac{\mathrm{p_b}(s|\mathbf{x})\mathrm{p_b^{(t)}}(\mathbf{x})}{\mathrm{p_b^{(t)}}(s)}\right] \quad . \tag{S2}$$

This is the iterative learning procedure used in our simulations for Fig 4.

The iterative procedure defined by equation ($S2$) has a fixed point in which the marginal likelihood on stimuli $\mathrm{p_b}(s)$ equals the experimental distribution of stimuli $\mathrm{p_e}(s)$, as we now show. A fixed point is reached when there is no change in the prior

from one iteration to the next, so that $\frac{\mathrm{p}_\mathrm{b}^{(t+1)}(\mathbf{x})}{\mathrm{p}_\mathrm{b}^{(t)}(\mathbf{x})} = 1$. Dividing both sides of equation (S2) by $\mathrm{p}_\mathrm{b}^{(t)}(\mathbf{x})$ gives

$$\begin{aligned}
\frac{\mathrm{p}_\mathrm{b}^{(t+1)}(\mathbf{x})}{\mathrm{p}_\mathrm{b}^{(t)}(\mathbf{x})} &= \mathbb{E}_{\mathrm{p}_\mathrm{e}(s,C)}\left[\frac{\mathrm{p}_\mathrm{b}(s|\mathbf{x})\mathrm{p}_\mathrm{b}^{(t)}(\mathbf{x})}{\mathrm{p}_\mathrm{b}^{(t)}(s)\mathrm{p}_\mathrm{b}^{(t)}(\mathbf{x})}\right] \\
1 &= \sum_C \mathrm{p}_\mathrm{e}(C)\int_s \mathrm{p}_\mathrm{e}(s|C)\frac{\mathrm{p}_\mathrm{b}(s|\mathbf{x})}{\mathrm{p}_\mathrm{b}^{(t)}(s)}\mathrm{d}s \\
1 &= \sum_C \mathrm{p}_\mathrm{e}(C)\int_s \frac{\mathrm{p}_\mathrm{e}(C|s)\mathrm{p}_\mathrm{e}(s)}{\mathrm{p}_\mathrm{e}(C)}\frac{\mathrm{p}_\mathrm{b}(s|\mathbf{x})}{\mathrm{p}_\mathrm{b}^{(t)}(s)}\mathrm{d}s \\
1 &= \int_s \mathrm{p}_\mathrm{b}(s|\mathbf{x})\frac{\mathrm{p}_\mathrm{e}(s)}{\mathrm{p}_\mathrm{b}^{(t)}(s)}\sum_C \mathrm{p}_\mathrm{e}(C|s)\mathrm{d}s \\
1 &= \mathbb{E}_{\mathrm{p}_\mathrm{b}(s|\mathbf{x})}\left[\frac{\mathrm{p}_\mathrm{e}(s)}{\mathrm{p}_\mathrm{b}^{(t)}(s)}\right]
\end{aligned}$$

If the marginal distribution of s in the brain's model at time t equals the experimenter's distribution on s, then the term inside the expectation is 1 and hence the brain has correctly converged to a model of the task.

The above demonstrates that the apparent circularity of equation (2) is in fact a feature of any "well-calibrated" probabilistic model. The fixed-point derivation above shows that when the marginal distribution of stimuli under the brain's (implicit) generative model matches the true distribution of stimuli defined by the experimenter, the process has converged and the relation in (2) holds. Alternative derivations of this relation can be found in the Methods section of the main text and in the Supplement of Berkes et al (2011) [1].

Note on relaxing the limits on the stimulus distribution

Our proof of (5) required a set of two limits in which (1) the stimulus distribution approaches a mixture of Dirac deltas at $s = 0$ and $s = \pm\Delta s$, and (2) the spread of these components becomes small, i.e. Δs gets small (but must not reach 0). These conditions might be considered extreme even for threshold psychophysics. In principle, this limits the applicability of our result whenever the empirical stimulus distribution has appreciable variance. In practice, however, three factors aid in the generality of our results. First, we have considered only the case where the forms a binary categorical judgment about, rather than an intermediate continuous estimation of the stimulus s. Even in two-alternative forced-choice tasks, subjects may internally categorize stimuli according to more than two categories, for instance distinguishing "faintly rightward" separately from "strongly rightward." To the extent that subjects *internally* make fine categorical distinctions such as this, our result concerns categorical beliefs about "faint" categories near the $s = 0$ boundary. This necessarily involves a small range of values of s around $s = 0$, as in the limiting case our proof requires. Another way to say this is that forming a continuous internal *estimate* of s that then informs the category judgment could be formalized as a limit where the number of fine-grained categories grows large. It is perhaps unsurprising that fluctuating internal continuous *estimates* of s elicit differential correlations when fed back as a prior and integrated with the true s. The limit required for our result for variable categorical beliefs can be interpreted as approaching continuous estimates around $s = 0$.

The second factor regarding generality is that the brain cannot represent arbitrary distributions, but is necessarily restricted to some finite approximation (whether by finitely many parameters in a parametric approximation, or finitely many values of $\mathbf{x}$ in a sampling-based approximation). Any family of approximations is a subspace of all possible distributions. Geometrically, one may think of "projecting" the true distributions $\mathrm{p}(\mathbf{x}|\ldots)$ into this subspace of approximating distributions. This projection operation will tend not to amplify differences between distributions, but will generally suppress them; the difference between approximate distributions will be less than the difference in the full space of distributions. Recall that in our derivations we used two distinct limiting processes: one where the entropy of each category shrunk (S1 Figb), and a second where their means moved towards zero (S1 Figc). After taking the first limit, the proportionality in (5) reduced to the question of whether $\mathrm{p}_{\mathrm{b}}(\mathbf{x}|\mathbf{E}(s=0))$ approximately equals $\mathbb{E}_{\mathrm{p}_{\mathrm{e}}(s)}\left[\mathrm{p}_{\mathrm{b}}(\mathbf{x}|\mathbf{E}(s))\right]$. While these terms may differ significantly in probability space, their projections may not. In other words, *the brain's distributional coding scheme may not be sensitive to these exact differences.* This suggests that the simpler the distributions represented by the brain the better our results will hold, since more distributions in the full space map to the same point in the subspace of approximate distributions when the approximating family is limited.

Third, note that our use of Dirac delta limits in $\mathrm{p}_{\mathrm{e}}(s)$ is, in part, an artifact of our mathematical approach. Because we placed very few restrictions on $\mathbf{x}$ and $\mathcal{R}$, we then required stronger constraints on $\mathrm{p}_{\mathrm{e}}(s)$ in order for an exact match between $\mathrm{d}\mathbf{f}/\mathrm{d}\pi$ and $\mathrm{d}\mathbf{f}/\mathrm{d}s$ to fall out. The precise way in which our derivation breaks down outside the sub-threshold regime is instructive: when $\mathrm{p}_{\mathrm{e}}(s|C)$ is wide, fluctuations in categorical belief ($\pm\Delta\pi$) result in changes to the posterior at values of $\mathbf{x}$ corresponding to a wide range of values of s, including some far away from the $s=0$ boundary. In other words, a wide prior means that changes to the prior can impact the "far away" values of $\mathbf{x}$, while changes to the likelihood are typically more "local." This initially seem to suggest that wider priors, formed through experience with a wide range of both sub- and supra-threshold stimuli, lead to larger deviations between the feedback direction and $\mathbf{f}'$. However, these deviations in the tails of the prior can be attenuated if the likelihood concentrates near $s=0$; a narrow likelihood suppresses variations in the prior in parts of $\mathbf{x}-$space corresponding to "large" s. To make such statements rigorous would require formalizing terms like "large" and "local." It seems plausible that one could carry out this analysis to a quantitative bound the amount of misalignment between $\mathrm{d}\mathbf{f}/\mathrm{d}\pi$ and $\mathrm{d}\mathbf{f}/\mathrm{d}s$ in terms of quantities like (i) discriminability of s (ii) heaviness of the tails of the likelihood, (iii) the experimental distribution of stimuli, and (iv) the relative (in)sensitivity of $\mathcal{R}$ to changes in higher frequencies of $\mathrm{p}_{\mathrm{b}}(\mathbf{x}|\ldots)$. We leave such further analyses to future work, but note that the spirit of our results may extend well beyond the strict assumptions we have made about the experimental distribution of stimuli.

Taken together, these points suggest that although the proportionality in (5) is approximate, its accuracy degrades gracefully under more realistic assumptions.

Derivation of (27) in terms of tuning to noise

If we approximate $\boldsymbol{\epsilon}$ as Gaussian, then from the Taylor expansion of $f_i(s=0,\pi={}^1\!/_2;\boldsymbol{\epsilon})$ around the mean noise value, it is easy to show that the covariance between neurons i and j due to noise is approximately

$$\mathrm{cov}_{\boldsymbol{\epsilon}}(f_i,f_j)\approx\nabla_{\boldsymbol{\epsilon}}f_i^{\top}\boldsymbol{\Sigma}_{\boldsymbol{\epsilon}}\nabla_{\boldsymbol{\epsilon}}f_j,$$

where $\boldsymbol{\Sigma}_{\boldsymbol{\epsilon}}$ is the covariance of $\boldsymbol{\epsilon}$, and $\nabla_{\boldsymbol{\epsilon}}f_i$ is the sensitivity of neuron i to variations in the noise around its mean. Computationally, the noise $\boldsymbol{\epsilon}$ acts on f_i through the intermediate step of the posterior, $\mathrm{p}_{\mathrm{b}}(\mathbf{x}|s=0,\pi={}^1\!/_2;\boldsymbol{\epsilon})$. Applying the chain rule, the

gradient of f_i with respect to $\boldsymbol{\epsilon}$ can thus be written as the product of f_i's sensitivity to $\mathrm{p_b}(\mathbf{x}|\ldots)$ and the derivative of $\mathrm{p_b}(\mathbf{x}|\ldots)$ with respect to $\boldsymbol{\epsilon}$. The chain rule gives $\nabla_{\boldsymbol{\epsilon}} f_i = \mathbf{J}_{\boldsymbol{\epsilon}}^{\mathbf{p}} \nabla_{\mathbf{p}} f_i$, where $\mathbf{J}_{\boldsymbol{\epsilon}}^{\mathbf{p}}$ is the Jacobian (i.e. columns of $\mathbf{J}$ are gradients of elements of $\mathrm{p_b}(\mathbf{x}|\ldots)$ with respect to the vector $\boldsymbol{\epsilon}$). The above covariance expression then becomes

$$\boldsymbol{\Sigma}_{ij}^{\boldsymbol{\epsilon}} \approx \nabla_{\mathbf{p}} f_i^\top \underbrace{\mathbf{J}_{\boldsymbol{\epsilon}}^{\mathbf{p}\top} \boldsymbol{\Sigma}_{\boldsymbol{\epsilon}} \mathbf{J}_{\boldsymbol{\epsilon}}^{\mathbf{p}}}_{\boldsymbol{\Sigma}_{\mathbf{p}}} \nabla_{\mathbf{p}} f_j \quad . \qquad \text{((27) restated)}$$

Thus we see that the covariance in neural responses induced by task-independent noise can be thought of in a two-step process: the covariance structure of the noise ($\boldsymbol{\Sigma}_{\boldsymbol{\epsilon}}$) induces correlated variability in the posterior density ($\boldsymbol{\Sigma}_{\mathbf{p}}$) through the Jacobian matrix of sensitivities ($\mathbf{J}_{\boldsymbol{\epsilon}}^{\mathbf{p}}$), which in turn manifests as correlated *neural* variability as per the "chain rule" argument from (1).

References

1. Berkes P, Orbán G, Lengyel M, Fiser J. Spontaneous Cortical Activity Reveals Hallmarks of an Optimal Internal Model of the Environment. Science. 2011;331(January):83–87.
