# Supplementary material for: Task-induced neural covariability as a signature of approximate Bayesian learning and inference
Source: PLoS Comput Biol. 2022 Mar 8;18(3):e1009557. doi: 10.1371/journal.pcbi.1009557 (PMC8963539; doi:10.1371/journal.pcbi.1009557)
Supplement: Attachment — Submitted filename: task_induced_cov_replies.pdf [file pcbi.1009557.s005.pdf]

Task-induced neural covariability as a signature of approximate Bayesian learning and inference: reply to reviewers

Reviewer 1

This paper analytically derived neural signature of tasks-specific signature by analyzing noise correlation and choice probability for 2-AFC task in a Bayesian probabilistic coding framework. The analytical results are supplemented by simulations when closed-form solutions were difficult to obtain. The paper also summarized previous experimental results that could be interpreted as evidence supporting the theory. Furthermore, the authors used a simple principal component analysis (PCA) method to show that the statistical structure of the neural responses could be used to infer internal belief about the stimulus in simulated/synthetic data.

Overall, I find this paper to be a valuable contribution to the field of neural coding. It contains various interesting ideas, which are likely to have an impact on the interpretation of noise correlations in the context of feedforward v.s. feedback processing. The analytical derivation under the Bayesian framework is elegant and thorough. There are some concerns I have with regard to the writing, along with some technical points, which need to be further clarified. Although these concerns slightly reduce the enthusiasm, I still consider this paper as a useful and timely addition to the literature.

Thank you for the thorough review and positive assessment.

One main concern of the current version is the readability. The paper is bit difficult to understand at this point. This is partly due to that many results were presented, but there seems to be a lack of focus. i) The predictions are scattered throughout the paper. The authors may consider summarizing the predictions explicitly somewhere, perhaps in the beginning of the Discussion.

Thank you for this suggestion. we have rewritten the end of the introduction and the beginning of the discussion to summarize the results more clearly.

ii) The first three sections of the Results are basically setting up the stages without actual results. I found them to be difficult to follow because too many concepts were introduced/defined. It would be useful if these could be streamlined and compressed.

Thank you for the feedback on readability. In the revised version we have tried to simplify where possible and added explanations. We have also combined the first two figures, so that there is now a single conceptual background figure, the running example of orientation discrimination is immediately introduced, and novel content now begins with Figure 2 (previously 3). Although Figure 2 (distinguishing two kinds of variability) is still "conceptual," making the difference between variability in the posterior and variability in the encoding explicit (rather than e.g. seeing the variability of the encoded posterior as a consequence of a noisy encoding) is an important idea

that is crucial for understanding the rest of the paper and will be new to most readers, so we have decided to leave it as-is, with only a few minor upgrades for clarity.

If the reviewer has specific suggestions about which parts s/he considers non-essential, we would be happy to delegate them to the Methods section or SI. We feel that clear definitions of how we use all terms, and in particular a clear separation of the computational level (Bayesian probabilities) and the empirically observable biophysical level (spikes and tuning curves) is one of the contributions of our paper, and essential for deriving our results. See, for instance, our preprint at https://doi.org/10.1101/2020.10.14.339770 on the different perspectives existing studies have taken when formalizing the idea of Bayesian inference in the brain, making it essential that we clearly lay out how we arrive at our analytical results.

Another more technical concern is the use of the term "differential correlation" which is used many times in the paper. In the original publication by Moreno-Bote et al (2014), "differential correlation" was defined based on the derivative of the tuning curve at each stimulus value. However, in the present paper, "differential correlation" is computed based on the difference between a pair of stimuli, i.e., $f(\theta_1) - f(\theta_2) = \delta f$. Although some recent studies have been making no distinction about the two, this does not seem to be a trivial distinction, because Fisher information at any stimulus value is related to the derivative of the tuning curve— this is defined in the absolute sense for that particular stimulus, not relative to another stimulus. Thus fundamentally, it is related to local discrimination, not general discrimination.

There may be a misunderstanding here since we are simply using the finite difference approximation that underlies the classic definition of a derivative. We may not fully understand this comment, but offer two potential clarifications in hopes that one of them addresses the present concern:

1. We use $\frac{f(\Delta s)-f(-\Delta s)}{2\Delta s}$ as an estimate of the tuning slope around $s = 0$. This is a good approximation as long as Δs is small or f is close to linear in the range between $-\Delta s$ and $+\Delta s$. In this sense, our use of $\pm\Delta s$ does not make the f' direction "relative to another stimulus," but is simply the common definition of f' at $s = 0$. Of course, taking the limit of $\Delta s \to 0$ gives the exact derivative. These linearity conditions are stated (somewhat indirectly) in the methods, where we say

   > Our only restrictions on $\mathbf{x}$ and $\mathcal{R}$ are that $\mathrm{p_b}(\mathbf{x}|\ldots)$ must change sufficiently smoothly with s, and $\mathcal{R}$ must be sufficiently smooth over the relevant range of stimulus values, so that the derivatives and linear approximations throughout are valid. A second restriction on $\mathbf{x}$ and $\mathcal{R}$ is that the dominant effect of s on $\mathbf{r}$ must be in the mean firing rates rather than their higher-order moments of $\mathbf{r}$. While this is a theoretically complex condition to meet involving interactions between s, $\mathbf{x}$, and $\mathcal{R}$, it is easily verified empirically in a given experimental context...

2. Neurons have many possible tuning curves with respect to many different stimulus parameters that are under experimental control. For instance, the same neuron could be analyzed in a fine-discrimination or coarse-discrimination context. Take fine orientation discrimination, where $s = 0$ is a vertical grating, in which case stimuli at $\pm\Delta s$ correspond to slightly left- or right-tilted gratings. The neuron's sensitivity to s in this context is the familiar definition of neurometric sensitivity to orientation around vertical. Now consider a coarse orientation discrimination task, where the image ($\mathbf{E}$) contrast is set by the magnitude $|s|$ and it switches between a vertical grating ($\mathbf{G}_0$) and a horizontal grating ($\mathbf{G}_{90}$) depending on the sign of s.

In other words,

$$\mathbf{E}(s) = \begin{cases} |s|\mathbf{G}_{90} & \text{if } s > 0 \\ |s|\mathbf{G}_0 & \text{if } s \leq 0 \,. \end{cases}$$

In this case, we would define $f(s)$ to be the tuning curve to this "contrast" variable s. The derivative f' could then be defined as above, using small values of $\pm\Delta s$, corresponding to the difference in response to a low-contrast vertical and low-contrast horizontal grating. Other coarse discrimination tasks follow a similar logic, e.g. with the classic dot-motion stimulus, the same argument applies but with "coherence" instead of "contrast." The relevant sense of "differential covariance" in these coarse discrimination tasks is the f' as we have just defined it, where the derivative is taken with respect to contrast (or coherence) at the discrimination boundary, i.e. at zero signal.

Relatedly, there is a sense that the noise correlation along the f'direction (f' represents the derivative of the tuning curve, not δf for an arbitrary pair) is specifical, because a small input noise (or stimulus noise) will naturally lead to a component in this direction. Therefore, I find the arguments around Fig. 6c to be unconvincing. In particular, the statement in lines 449-452 ["This makes the f' direction largely arbitrary, so the results of Rabinowitz et al. (2015), as well as other recent findings of alignment between f' and the low-rank modes of covariance (Bondy et al., 2018; Montijn et al., 451 2019; Rumyantsev et al., 2020), ought to be extremely surprising in a feedforward framework or in models of low-rank but task-independent variability"] is quite problematic in my view due to the impact of shared input noise. The same statement was basically repeated in line 552-554. I don't see why this should be considered as "extremely surprising" as claimed in the paper. In general, statements like this are perhaps too subjective to be useful.

In an effort to clarify, we have elaborated some in the text at the lines that you have highlighted here, and have added text near equation (7), which introduces the terms $\Sigma^{\text{intrinsic}}$ and Σ^{belief}. We agree that afferent noise contributes to differential correlations through $\Sigma^{\text{intrinsic}}$, but that is true for all directions in the space spanned by the input dimensions (Kanitscheider et al. 2015) without privileging the single direction defined by the specific experimenter-defined task in a given experiment.

Let us expand, and explain in more detail why our statements are not "too subjective to be useful." Your point about shared input noise is also made by Kanitscheider et al (2015), who simulate a population of V1 neurons, including upstream noise in both the retina and LGN. Their simulation revealed significant nonzero differential correlations in the V1 population (for fine orientation discrimination around horizontal), which indeed *must* be present since upstream information is limited. However, limited input information means that there must be differential correlations with respect to any other task as well, including fine discrimination tasks around *all* other reference orientations, coarse discrimination, and others, and each of these will further depend on other properties like the spatial frequency of the stimulus. Crucially, the studies we cite above, like that of Rumyantsev et al. (2020), do not simply find "nonzero" differential correlations (which we agree would be unsurprising and uninteresting), but that differential correlations *with respect to the particular task that the animals learned in their experiment* were larger than almost any other direction of variance in the space. The relevant quantitative question is this: in a high-dimensional neural space, what is the span of all possible $\mathbf{f}'$ vectors for all possible tasks? This span is of the order of the dimensionality of the inputs to the neural population that is being recorded from, making the probability vanishingly small that the variability is largest for the $\mathbf{f}'$ direction chosen arbitrarily for any one study.

Regarding the generality of the results: the results in the paper are limited to the 2-AFC task. The continuous estimation problem is only briefly discussed in SI (line 1259-1273). It would be useful to bring the discussions on continuous estimation problem up to the main text. This will help partly address the question of generality. Also, Fisher information (and differential correlation) comes naturally with the continuous estimation problem.

We have focused our derivation and results on discrimination tasks which underlie the vast majority of perceptual decision-making studies for which neural responses are available. We agree that continuous estimation is interesting, and believe that our formalism and results can straightforwardly be extended to most estimation tasks. As long as there is a one-dimensional stimulus s, giving rise to a range of sensory inputs $\mathbf{E}(s)$, then the average posterior $p(\mathbf{x}|\mathbf{E}(s))$ will have most of its mass along the corresponding trajectory in latent $\mathbf{x}-$space, and hence neural $\mathbf{r}-$space, and so will the top-down prior after learning. As a result, variability in the mean of π (which is now a distribution over s) will induce differential correlations in the $\mathbf{f}'-$direction. However, we feel that making the above sketch of a proof as rigorous as the rest of our paper would require additional work that would go beyond the scope of an already long paper. We now include in the last discussion paragraph a short version of the argument previously in the SI.

Specific comments:

* Line 19-20. It is stated that the predictions of the proposed framework differ from the ones form the feedforward models. Reading through the paper, I am not exactly sure what these differences are. It would be useful to summarize these explicitly.

To clarify this, we have elaborated the first two paragraphs in the section titled "Connections with empirical literature." Briefly, our predictions for the relationship between choice probabilities and neural sensitivity agree with feedforward optimal linear decoding (both predict $CP \propto d'$), though they differ in underlying mechanism, which could in principle be tested empirically in the future through causal interventions on the feedforward or feedback pathway. Our *unique* predictions, that disagree with those from a purely feedforward framework, is the task-dependence and learning-dependence of correlated neural variability.

* Line 39-41 stated that the results hold under general assumptions. However, later it was shown that, when there is noise, the results only hold in some cases. There appears to be a discrepancy between what was claimed here (as well as lines 118-120) and the actual results. One of proof is based on taking the limit in another limiting regime, which is delicate. It would be helpful to re-write these sentences to reflect the subtlety of the results.

Thank you for pointing this out. We have now rewritten the last paragraph of the Introduction and the first paragraph of the Discussion, to more explicitly lay out which assumptions lead to which results. Our motivation for using the "generality" term was the fact that our results abstract away from particular hypotheses about internal models and distributional codes. But of course, the assumptions we do make are important, so we have now removed or reduced any "generality" claims throughout the manuscript.

* Could the authors clearly state the assumptions underlying the "fundamental self-consistency relationship", i.e. "the average posterior equals to the prior"? An average reader might not be familiar with this relationship.

The self-consistency relationship is stated in equation (2), where we have now elaborated a bit further on its origin. We have also added a short derivation at the beginning of the Methods

section. It is an immediate consequence of the rules of probability, $p(y) = \int p(y|x)p(x)\,\mathrm{d}x$ – see e.g. the supplementary information of Berkes et al. 2011 (S42) or the textbook by Dayan and Abbott (2001) which states, on page 364,

> The recognition model provides a mechanism for checking the self-consistency of [the generative model]. This is done by examining the distribution of causes produced by the recognition model in response to actual data. This distribution should match the prior distribution over causes... If the prior distribution of the generative model does not match the actual distribution of causes produced by the recognition model, this is an indication that the [model] does not apply accurately to the input data.

Neither of these texts provide further references either.

* "differential correlation" needs to be explicitly and clearly defined (also see my comments earlier).

We have added a paragraph defining them at the start of the section titled "Feedback of variable beliefs implies differential correlations."

*Line 534-536 stated that "some" of the measured "differential" correlation could be understood as near-optimal feedback. I found this description to be rather vague. What if the feedback is not near-optimal? In that case, would some "differential" correlation still be predicted? If the answer is yes, then the interpretation of "near-optimal feedback" based on "differential" correlation would not be valid.

We have now rephrased this line. Previously, differential correlations were taken to be identical with information-limiting, based on a feedforward source. In this paragraph, we discuss the new insights from our Results that tell us that this is not necessarily so, and under what situations the feedback-induced part of the differential correlations limits information or does not. Our focus in the present paragraph (now starting on Line 620) is to address whether an increase in differential correlations implies a reduction in information.

* Line 522-524. The connection to the recurrent neural networks is not clearly described.

We have now clarified it as "models of "state-dependent" recurrent dynamics". Our point is simply that a feedback prior that shapes internal variability is akin to feedback-induced states that shape the recurrent dynamics as previously proposed in a more mechanistic framework.

* For the clarity of the presentation, it would be useful to label the subsections of Method for the sake of referencing in the main text. Currently, it is difficult to locate the proof for a particular statement stated in the main text.

Thank you for this excellent suggestions – we have incorporated it in the revised version!

* Would it be useful to fit a regression line in Fig. 6a? That might help better convey the point.

This is an excellent idea, and we have now updated the plot accordingly.

* It is difficult to see the connection between Fig. 6b and the claims made in Line 439-443, and why Fig 6b is supporting the authors' claim. Could the authors please unpack the connection there?

Thank you for pointing out the gap in our explanations there. We have now expanded on the link between the results of Rabinowitz et al (2015) and our predictions, both in the caption and in the text.

* Line 334-349 describes a special class of encoding schemes for which the results (Eq. 8 and Eq. 9) still hold when the noise is present. Is this class of code both necessary and sufficient? It is obviously sufficient based on what is described there, but unclear whether it is also necessary. For the prediction in Line 347-349, I'd think one would need the linearity to be a necessary condition as well. If it is both necessary and sufficient, it would be useful to state that explicitly. If it is not, it would be useful to tone down the claim in the last sentence.

This is an interesting question that we had not considered. We cannot claim necessity of LDCs in general, since we have imposed so few restrictions on $\mathbf{x}$, $\mathcal{R}$, and the task. This leaves open the possibility for, essentially, linear coincidences, or operating in a locally linear piece of a globally nonlinear code. We have now qualified the final sentence of this paragraph as suggested by the reviewer and added clarifications on necessity versus sufficiency in the Methods.

*Line 774-775 is difficult to understand. "map" or "mapping"?

Thank you! We meant mapping, but have now simply omitted this non-essential sentence.

*Line 182- 187. These lines are difficult to follow.

Thank you for alerting us to this. We have now rephrased this paragraph to make it clearer and easier to follow.

*Fig 1d. I found the scheme is confusing, in particular the direction of arrows. Maybe I don't quite understand what this figure is trying to show.

Thank you for pointing out that the meaning of the arrows wasn't clear. We have now removed this figure panel as part of our efforts to streamline the first sections. In the new figure, we have now made explicit that black arrows represent statistical dependencies (the conditional independence of standard Bayesian networks), and the blue and red arrows represent the information flow associated with likelihood and prior.

We have now also combined figures 1 and 2 in order to combine all figure related to the setup for our study, and not representing new insights. The intention of Figure 1 is to illustrate (a) the concept of generative models (the now-removed "car" example had been added only for redundancy on this point), (b) introduce our notation for the key variables, and (c) illustrate the links between the abstract computational level of description and the empirical level at which the experimental observations are made.

*Line 110: "two frameworks". Not sure if it would be accurate to call the studies on noise correlation as a "framework"—perhaps more fair to use "two branches of research"?

Thank you for pointing out this potential confusion. We have now clarified that we are referring to the Bayesian inference framework, and the signal processing framework.

Reviewer 2

The manuscript discusses the effect of probabilistic inference on neural response statistics. In particular, the focus of the paper is inference of task-related variables on the covariation of neural responses. The paper establishes normative relationship between distinct neural phenomena, such as top-down feedback, choice probabilities and information limiting correlations. Besides the appeal that a formal treatment is provided to higher order response statistics, a further aspect that helps appreciating the theory is that it discusses alternative computational approaches jointly. The paper provides important insights into the way computation-level considerations are linked to neural population activity.

The paper is very dense but is clearly written and arguments are well supported by formal analysis. I only have a number of minor clarification suggestions/questions.

Thank you for the overall positive assessment and helpful suggestions.

Questions: I have one question that might require some additional writing. In the paragraph starting at line 260 the authors lay out the requirements for their analytical treatment. The assumptions are sound and adequate for the particular setting they analyze but the authors would benefit from a discussion of how these assumptions affect the generality of the claims. For instance, while a fully learned model can be assumed but some qualitative aspects of the model would still hold even if there is a discrepancy between the internal model and the actual model of the task. Importantly, there are tools to infer variations in the strategy followed by animals from behavior alone, therefore one could expect these changes to be captured by the theory presented here. From an experimental point of view, the second assumption is certainly true for the analyzed experiments but the basic consequences of the theory are more general than stimuli presented close to the psychometric threshold.

Both you and the first reviewer raised a similar concern about the "generality" of our results. We had intended for this to refer to the fact that our results make very few assumptions about the nature of distributional codes in the brain (e.g. sampling or parametric), but we appreciate that claims of "generality" are tempered by the rather specific style of sub-threshold 2AFC task we analyze. In our revised version, we've made numerous changes throughout to address this.

We also appreciate the suggestion to analyze behavior as a separate verification or source of predictions. Indeed, if a subject's internal model of the task differs from the true task, then this can often be gleaned from their behavior (e.g. by reverse-correlation methods) and our theory would predict that noise covariance has a component that aligns with the subject's (mistaken) beliefs about the task. In other words, the relevant direction in neural space would be $\mathbf{f}'$ with respect to the stimulus categories the subject *thinks* they are discriminating. A similar point is illustrated in our PCA plot (now Fig 6), where the simulated neural data reveals the model's uncertainty about which of two tasks it is doing, as well as in the discussion paragraph on "the three possible deviations from our assumptions." We've now included brief comments about behavioral methods in both places.

We especially appreciate the comment that "the basic consequences of the theory are more general than stimuli presented close to the psychometric threshold." This prompted us to revisit exactly why each of the four assumptions is needed, and why. We were, in fact, a little surprised when we first developed assumptions 2 and 3 in our research, as we had hoped initially to formalize our intuitions about categorization tasks more generally, which in practice often include non-negligible numbers of supra-threshold trials. Upon revisiting this, we realized we could add a third major argument in favor of "graceful degredation" from our predictions to the Supplemental

Text. We include a copy of the new Supplemental Text paragraph here:

> Third, note that our use of Dirac delta limits in $\mathrm{p_e}(s)$ is, in part, an artifact of our mathematical approach. Because we placed very few restrictions on $\mathbf{x}$ and $\mathcal{R}$, we then required stronger constraints on $\mathrm{p_e}(s)$ in order for an exact match between $\mathrm{d}\mathbf{f}/\mathrm{d}\pi$ and $\mathrm{d}\mathbf{f}/\mathrm{d}s$ to fall out. The precise way in which our derivation breaks down outside the sub-threshold regime is instructive: when $\mathrm{p_e}(s|C)$ is wide, fluctuations in categorical belief ($\pm\Delta\pi$) result in changes to the posterior at values of $\mathbf{x}$ corresponding to a wide range of values of s, including some far away from the $s = 0$ boundary. In other words, a wide prior means that changes to the prior can impact the "far away" values of $\mathbf{x}$, while changes to the likelihood are typically more "local." This initially seem to suggest that wider priors, formed through experience with a wide range of both sub- and supra-threshold stimuli, lead to larger deviations between the feedback direction and $\mathbf{f}'$. However, these deviations in the tails of the prior can be attenuated if the likelihood concentrates near $s = 0$; a narrow likelihood suppresses variations in the prior in parts of $\mathbf{x}-$space corresponding to "large" s. To make such statements rigorous would require formalizing terms like "large" and "local." It seems plausible that one could carry out this analysis to a quantitative bound the amount of misalignment between $\mathrm{d}\mathbf{f}/\mathrm{d}\pi$ and $\mathrm{d}\mathbf{f}/\mathrm{d}s$ in terms of quantities like (i) discriminability of s (ii) heaviness of the tails of the likelihood, (iii) the experimental distribution of stimuli, and (iv) the relative (in)sensitivity of $\mathcal{R}$ to changes in higher frequencies of $\mathrm{p_b}(\mathbf{x}|\ldots)$. We leave such further analyses to future work, but note that the spirit of our results may extend well beyond the strict assumptions we have made about the experimental distribution of stimuli.

In the section 'Variable beliefs in the presence of noise' the authors argue that the alignment of $\mathrm{d}f/\mathrm{d}s$ and $\mathrm{d}f/\mathrm{d}\pi$ is critical for the derivations to hold and then they introduce LDC's that can overcome theoretical hurdles. This fine theoretical insight is proposed to be a basis for experimental validation. While this is indeed true, additional insights would be welcome if this is something that can indeed be measured in electrophysiological data.

Reviewer 1 similarly requested clarification on necessity or sufficiency of the LDC assumption. We have now very briefly expanded on the role of the linearity assumption and the kinds of predictions one could make about linear versus nonlinear codes. However, we feel it is outside the scope of this already large paper to dive more deeply into the properties and predictions of LDCs.

The authors argue that the self-consistency claim that the results are based on are general across theories of probabilistic computations. The argument for this claim is properly spelled out. It would be instructive for the reader to briefly discuss if these theories are indistinguishable in the discussed context.

By "theories of probabilistic computations" we assume you mean algorithms like sampling, PPCs, DDCs, etc. We tentatively agree that they are indistinguishable *in the discussed context*, keeping in mind that our context was deliberately restricted. We hope that the revised version, especially in the introduction and first section of results, clarifies that our results are deliberately agnostic to the "encoding of a given posterior," which could contain signatures that distinguish different distributional coding theories.

clarifications:
line 110: I suggest spelling out the 'two frameworks': The reader might lose track by this time

Great suggestion – implemented in the revision.

Caption of fig 2: s is not defined in time to understand the figure.

This is now combined with Figure 1, where the caption now introduces s with the example of an oriented grating.

Caption of fig 2: 'Our derivation assumes that smoothly changing posteriors (a) corresponds to smooth changes in neural responses' -¿ one could introduce f(s) here since later f' turns up in the caption.

Many thanks for this good suggestion which we have incorporated in the revision. line 149: 'variability of posterior distributions' is not sufficiently motivated at this point

Thank you for pointing out this mistake: we meant to say 'structure in the posterior'.

Caption of fig 3: 'Variability in the underlying posterior may appear as correlated variability in spike counts.' -¿ I appreciate the argument but I am not sure how the figure motivates this point.

Thank you for pointing out the need to better explain the figure. We have now expanded the caption to address this.

line 255: please explain $\pi = \frac{1}{2}$

Thank you for pointing this out. We had implicitly assumed an unbiased observer who's belief about the correct category is 50-50, i.e. $\pi = 0.5$, at the decision boundary ($s = 0$). We have explained this better in the text.

line 274: Changes induced by top-down influences affect the tuning curve. It would be useful to note how this affects measuring tuning curves

This is an interesting and helpful note. We have to answers, depending on whether we are thinking in terms of long-term or short-term changes to tuning.

Consider long-term changes over learning. By definition, a tuning curve is the average response as a function of some experimenter-chosen s. The act of averaging marginalizes out the top-down influences on $\mathbf{r}$. Since task-learning implies a changing prior over $\mathbf{x}$, and hence a changing posterior over $\mathbf{x}$, and since $\mathbf{r}$ is assumed to represent the belief over $\mathbf{x}$, task-learning in general will also affect tuning curves. A simplistic way of thinking about it is that neurons are really tuned to the brain's belief about s, not the experimenter-defined s directly, and that belief will in general be different after learning. Understanding the details of those changes depending on the particular task might be interesting but would go beyond the scope of the current paper. However, we do not believe this to be an important issue in our context since in the vast majority of neurophysiological studies relevant to our work, tuning curves are measured in temporal proximity (typically the same day) as correlations and choice-related signals. However, we do acknowledge that modern chronic recording techniques allow for holding individual neurons over increasing times, such that the question that the reviewer appears to be aiming at might become of empirical relevance soon.

For short-term changes (e.g. within a session), it is also worth noting that in cases where $df/ds \propto df/d\pi$, we would predict that small changes in s result in small changes to π *on average*, which would then reciprocally feed back to and selectively amplify the responses of neurons with positive f' and suppress those with negative f'. The result in the population would be a bias in the sensitivity turning f' into $f' + cf'$ for some constant c. So, this would affect magnitude but

not the direction of f'. This is essentially the argument we lay out in the methods surrounding equation (15). Accounting for changes to π driven by s would give the *total* derivative, but due to the redundancy of the two terms, we only need to consider the *partial* derivative with respect to s.
